# Who Buys Surplus Meals? An Exploratory Survey in Danish Canteens

**DOI:** 10.3390/foods12051035

**Published:** 2023-02-28

**Authors:** Sujita Pandey, Mausam Budhathoki, Kaixin Feng, Marianne Thomsen, Helene Christine Reinbach

**Affiliations:** 1 Department of Food Science, University of Copenhagen, Rolighedsvej 26, 1958 Frederiksberg C, Denmark; 2 Institute of Aquaculture, University of Stirling, Stirling FK9 4LA, UK

**Keywords:** consumer behaviour, food waste, surplus meal, theory of reasoned action, food-related lifestyle, sociodemographic characteristics

## Abstract

Food waste has received increasing attention over the last decade, owing to its economic, environmental, and social impacts. Much of the existing research has investigated consumers’ buying behaviour towards sub-optimal and upcycle food, but surplus meal buying behaviours are poorly understood. Thus, this study performed consumer segmentation through a modular food-related lifestyle (MFRL) instrument and determined consumers’ buying behaviour towards surplus meals in canteens employing the theory of reasoned action (TRA). A survey was conducted using a validated questionnaire from a convenient sample of 460 Danish canteen users. Four food-related lifestyle consumer segments were identified by employing k-means segmentation: Conservative (28%), Adventurous (15%), Uninvolved (12%), and Eco-moderate (45%). The Partial Least Square Structural Equation Modelling (PLS-SEM) analysis indicated that attitudes and subjective norms were significantly influencing surplus meal buying intention to further influence buying behaviour. Environmental objective knowledge was significantly influencing environmental concerns to further influence attitudes and behavioural intention. However, environmental objective knowledge had no significant influence on attitude towards surplus meals. Male consumers with higher education, those having higher food responsibility and lower food involvement, and convenience scores had higher surplus food buying behaviour. The results can be used to inform policymakers, marketers, business professionals, and practitioners to promote surplus meals in canteens or similar settings.

## 1. Introduction

The complexity of food waste management and its impact on sustainable development has received increasing attention over the last decade [1,2,3,4]. Starting from the agricultural stage until the consumption stage, food loss and waste occur in every stage of the food supply chain (FSC) [5]. At present, food waste is responsible for around 8–10% of global greenhouse gas emissions and in the context of national emissions, it would be the third largest emitter, first being China (21%) and second being the United States (13%) [6,7,8,9].

The Food and Agriculture Organization (FAO) estimated that food waste and loss are responsible for a direct economic cost of about $1 trillion, increasing to $2.6 trillion when social and economic losses are also considered [10]. A recent study has shown that a 1% decrease in food waste is significantly associated with a reduced poverty of about 0.87% [11]. Moreover, the total amount of food waste generated per capita per day globally accounts for 18 daily healthy diets contributing a huge quantity of nutrients wasted with over 800 Kcal of energy wasted per person per day [12,13,14].

While billions of tonnes of quality edible foods are being wasted in high-income countries due to excess consumption, low-income countries on the other hand are combating nutritional deficiencies [15,16]. According to the FAO, both high and low-income countries account for an almost similar amount of food waste, nevertheless, the waste in low-income countries is a result of insufficient food-chain infrastructure is mostly harvesting and processing [2,17]. While the food waste generated in high-income countries is mostly at the retail and consumer level, mainly due to consumer behaviour [2,18]. There have been several studies to understand consumer food-waste behaviour and interventional studies in households [19,20,21], retail [22,23,24], and out-of-home dining settings [25,26,27]. The findings from these studies indicated that a multitude of factors influences consumers´ food-waste behaviour but fundamentally are underpinned by societal factors (e.g., socio-cultural and environmental factors), behavioural factors (e.g., habits and practices) and personal factors (e.g., sociodemographic and psychological factors) [28,29].

Another key element to tackling food waste generation is by reducing and recovering surplus foods as it is eatable food suitable for human consumption [5]. The food waste hierarchy highlights avoiding food surplus throughout the food production and consumption system to prevent food waste and reuse surplus food [30,31]. The reduction in surplus food can not only rescue the nutrients but also recovers all inputs such as energy, water, land, and fertilizers use and other costs associated with its production that would otherwise have been wasted; therefore, it is a win-win strategy [32,33]. Therefore, multiple strategies have been implemented to prevent or recover surplus foods from being wasted. For instance, price reduction, portion size reduction, smart packaging, food donations, schemes to recycle food and reduce food waste in the food and service sector (ReFood label), and Too good to go (a mobile app) have been adopted for surplus food management [34,35,36].

Recent data shows that food waste per capita for out-of-home consumption in Denmark was 21 kg and approximately 33 thousand tonnes of food waste is generated by Danish canteens annually [7,37]. In Denmark, one-third of the total food is consumed in canteens. While the majority of the Danish canteens sell their meals buffet style resulting in unwanted food waste generation [37,38]. Thus, the Stop Wasting Food movement (in Danish “Stop Spild Af Mad) had been launched in 2008, which has been a providing range of resources and tools to reduce food waste in Danish canteens including guides on menu planning, portion control, and food storage [39]. Meanwhile, some of the Danish canteens have implemented new practices, such as selling surplus meals marked as student dishes at a reduced price. The canteens are also adapting different food waste reduction strategies that are currently implemented in the other out-of-home dining settings, for instance, redesigning choice architecture, nudging, and price promotion strategies. Some of the Danish Canteens have also been effectively communicating food waste reduction strategies through social media and menu boards [40].

However, the success of these strategies in Danish canteens greatly depends on understanding consumers’ actual or anticipated surplus meal perceptions and surplus meal buying behaviours. To our knowledge, there have not been any studies aimed at understanding consumers´ buying behaviour towards surplus meals. Existing research has only investigated consumers´ buying attitudes and intentions towards suboptimal [41,42,43,44] and upcycled food [45,46,47,48]. The authors of [49] found that higher amounts of food waste were associated with young consumers. University/workplace canteens are considered important out-of-home eating environments for young consumers, where they buy a substantial amount of food and these settings are prone to food waste [50,51]. Due to a lack of literature focus in canteen settings, it is a challenge to develop effective marketing strategies to reduce food waste, for instance, by reducing surplus food generation. To fill this research gap, this study employed the extended theory of reasoned action to understand consumers’ buying behaviour towards surplus meals in canteens. The study also identifies Danish consumer segments to identify important characteristics for buying surplus meals.

## 2. Conceptual Framework and Hypotheses

The theory of Reasoned Action (TRA) is a conceptual framework that has been broadly applied in behavioural research on human action. The framework argues that human behaviour is predicted by behavioural intention and that intention is determined by attitudes and subjective norms (social pressure to perform the behaviour) [52]. Fishbein and Ajzen [53] argue that people select a reasoned option from a variety of available options. A previous meta-analysis confirms the predictive power of the TRA framework and highlights that in most situations TRA successfully predicts human behaviour, given that the behaviour is voluntary [54].

The study has adapted the framework to predict intention to purchase surplus meals, where the attitude reflects a person’s perspectives on purchasing surplus meals and subjective norms involve understanding the influences of their family and peers to purchase surplus meals. Previous studies conducted among Danish consumers have shown a positive attitude, subjective norms, and intention toward the rescue of food and the reduction in food waste [49,55,56]. Thus, the following hypotheses are generated.

**H1.**  *Attitudes towards purchasing surplus meals have a positive impact on the behavioural intention to buy surplus meals.*

**H2.**  *Subjective norms towards purchasing surplus meals have a positive impact on the behavioural intention to buy surplus meals.*

**H3.**  *Intention has a significant positive impact on buying surplus meals.*

Though it is evident that TRA has a strong validity; however, it is limited when it comes to predicting all types of human behaviour [54,57]. Previous studies have suggested enhancing the model’s explanatory power by modifying the original TRA by including additional variables [57,58]. The most reported variables that have been added previously to the model include perceived behavioural control [25,59,60] to predict food waste reduction behaviour. The addition of perceived behavioural control to TRA is considered the theory of planned behaviour (TPB) and TRA is a special case of TPB. TRA assumes that consumers have volitional control over the behaviour of interest (such as reducing food waste behaviour). Furthermore, behavioural control was difficult to access as some canteens do not have the option to buy surplus meals. Based on these assumptions, TPB to TRA was not considered appropriate for this study. This study rather included environmental objective knowledge, environmental concern, MFRL factors, and sociodemographic and lifestyle factors, which were better suitable for predicting surplus meal buying behaviour. Further, we selected these variables based on their practical value to policymakers, marketers, business professionals, and practitioners as the findings may easily be incorporated into their strategies to promote behavioural change in a specific consumer segment.

It is evident that consumers’ attitudes are shaped by environmental knowledge that influences their behaviour resulting in attitude-behaviour diversity [61,62]. Previous studies have highlighted that knowledge of environmental impact can positively affect consumers’ attitudes toward environmentally friendly products [63,64,65]. Moreover, a study by [66] showed that consumers’ positive or negative attitude towards environmentally friendly products is highly determined by the level of environmental knowledge. Besides the attitude, individuals’ environmental knowledge further shapes their concerns towards the environment and guides them to perform a certain action [67]. Thus, the study hypothesises.

**H4.**  *Environmental objective knowledge significantly affects attitudes to buying surplus meals.*

**H5.**  *Environmental objective knowledge significantly affects environmental concern to buy surplus meals.*

According to Bamberg [68], environmental concern guides situation-specific attitudes to perform specific environmental behaviours. Studies conducted previously have shown that individuals with higher environmental concerns had a positive impact on consumers’ attitudes toward purchasing green and environmentally friendly products [69,70,71]. Further, consumers had a higher intention to purchase sustainable foods when they had a higher level of environmental concerns [65,72,73]. Thus, the following hypotheses are generated.

**H6.**  *Environmental concern significantly affects attitudes to buy surplus meals*

**H7.**  *Environmental concern significantly affects the intention to buy surplus meals.*

The study extends the TRA framework with the concept of modular food-related lifestyle (MFRL) instruments to address such fundamental factors to get an insight into consumers’ food perception and personal values [74]. With growing interest in the ethics and sustainability of food in recent years, the concept of MFRL was proposed to conduct a basic segmentation of consumers according to their food-related lifestyle [75]. Brunsø and colleagues [73] argue that a modular approach to measuring food-related lifestyle mediates between life values and food-related behaviour. Thus, the three core dimensions namely, food involvement, food responsibility, and food innovation with some add-on dimensions depending on the aim of the study can retain the original means-end approach to food-related lifestyle.

Convenience is one of the major food waste drivers among Western consumers [76]. A previous study by [77] employed 24 food-related lifestyle factors to identify five segments, of these, two segments were characterised by a high share of convenience food consumption and food waste behaviour. A previous study has shown that convenience-oriented consumers are more willing to buy value-added surplus products [78]. Thus, based on MFRL and previous studies the following hypotheses are generated.

**H8.**  *MFRL factors (Involvement, Innovativeness, Responsibility, and Convenience) have a positive impact on the attitude to buy surplus meals.*

**H9.**  *MFRL factors (Involvement, Innovativeness, Responsibility, and Convenience) have a positive impact on behaviour to buy surplus meals.*

According to Glanz and colleagues [79], an individual’s attitude and subjective norms are influenced by their sociodemographic and lifestyle determinants creating an indirect intention to perform a certain behaviour. Multiple previous studies have investigated the influence of sociodemographic and lifestyle factors on consumers’ attitudes and subjective norms towards sustainable food buying behaviour [73,80,81]. Moreover, the results from previous studies show that age [80], gender [73], living status [82], education [73,80,83], employment [84], and income [73,80,83] are associated with the purchase of sustainable foods. Therefore, this study intended to examine the effect of socio-demographic and lifestyle factors on attitudes and subjective norms as well as surplus meal buying through the following hypothesis:

**H10.**  *Sociodemographic and lifestyle factors significantly affect attitude, to buy surplus meals.*

**H11.**  *Sociodemographic and lifestyle factors significantly affect subjective norms for buying surplus meals.*

**H12.**  *Sociodemographic and lifestyle factors significantly affect behaviour to buy surplus meals.*

**Figure 1:**
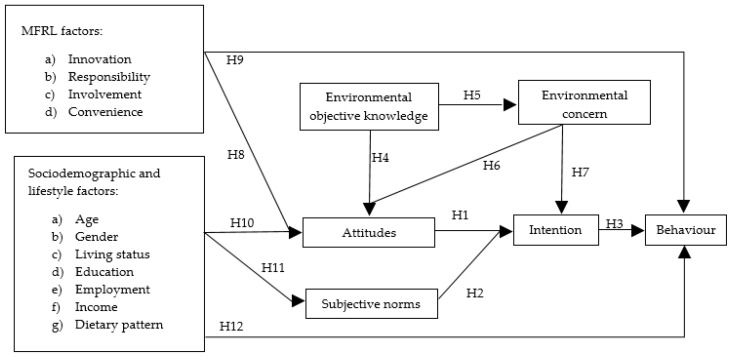
A conceptual framework based on TRA-extended model and testing hypotheses.

Based on the above, a proposed framework based on TRA-extended model (Figure 1) has been developed.

## 3. Methods and Materials

### 3.1. Questionnaire and Measurement Scale

The questionnaire was developed in English and administered in both English and Danish languages. The Danish version was translated by an independent translator and reviewed by four food experts from the Department of Food Science, University of Copenhagen, as well as eight Danish consumers. After the consensus of translation and review, a pre-final version was prepared, which was then pre-tested among 15 Danish consumers to check for consistency, layout, and readability.

The questionnaire was organised into four sections. The first part consisted of sociodemographic and lifestyle characteristics of the participants, including age (in years), gender (male, female), living status (alone, with a partner, with partner and children, single parent, living at home with parents, living with roommates, other), education (primary school, high school, vocational training, professional bachelor, bachelor, master, PhD, other), employment status (student, a student with a job, part-time job, full-time job, self-employed, unemployed, other), income (<100,000 DKK, 100,000 to 249,999 DKK, 250,000–499,999 DKK, 500,000 to 649,999 DKK, >650,000 DKK) and dietary pattern (omnivore, flexitarian, vegetarian, vegan, other).

In the second section, the psychographic characteristics of consumers were explored through MFRL dimensions including food involvement (2 items), food responsibility (2), food innovation (1), convenience (2), and price (1). The statements on FRL dimensions were assessed using a seven-point Likert scale ranging from 1 as “strongly disagree” to 7 as “strongly agree”. Thus, the selection of the MFRL dimensions and their items was inspired by previous studies on consumer food waste reduction behaviour [62,77,85] with adjustments that are relevant to the aim of this study.

In the third section, consumers reported their degree of agreement with the items measuring constructs of the proposed TRA-extended model (attitudes, subjective norms, environmental objective knowledge, environmental concern, intention, and behaviour). Behaviour (buying surplus meals) was based on the self-reported buying of surplus meals with the frequency of buying measured by the following item: “How often do you buy surplus meals at the canteen?”, ranging from 1 as never, to 5 as daily.

The final section consisted of consumers stated buying preferences for surplus meal types with options that included meat-based, plant-based, no preference, and I don’t want to buy a surplus meal. The reason for their decision to prefer surplus meal types was measured with 20 statements that include 4 statements for meat-based, 5 for plant-based, 4 for no preference, and 7 for I don’t want to buy a surplus meal.

Table 1 briefly shows the items measuring the MFRL instrument, constructs of the proposed TRA-extended model, and 20 reason statements as well as their source of adoption. Both the FRL items, the proposed TRA-extended model items and reasons for surplus meal preference were assessed using a five-point Likert scale ranging from 1 as “strongly disagree” to 5 as “strongly agree”.

### 3.2. Data Collection

Initially, the required sample size for the empirical study was determined through WarpPLS software that suggested a minimum sample size to estimate the path coefficient in the partial least square structural equation model of 0.15 at a significant effect level 0.05 with a power of 0.94 was 455 based on the inverse square root method and 438 based on the gamma-exponential method [94]. Thus, a convenient sampling technique was employed to recruit 498 participants. After excluding 38 incomplete responses, the final sample consisted of 460 Danish consumers. Data was collected through a web questionnaire in the Survey-Xact platform that was distributed on social media using a hyperlink to the questionnaire from 28 April to 9 May 2022. Further, posters were attached in the dining areas of several university/workplace canteens with a description of a project consisting of a QR code and a hyperlink. Canteen users between the age range of 18–65 years were included. Before completing the survey, written informed consent was obtained from each participant and were made aware of the time needed (approximately 5–10 min) to complete the survey. The study was conducted following the Declaration of Helsinki. All procedures involving study participants were approved by the Research Ethics Committee of Science and Health, University of Copenhagen (Ref: 504–0327/22–5000).

### 3.3. Data Analysis

The IBM SPSS Statistics version 28.0 was used for the data management and analysis [95]. Initially, responses to the three environmental objective knowledge statements were re-coded as 1 for correct answers and 0 for the wrong answer and the ‘I do not know’ response. The final environmental objective knowledge measure was computed as the total number of correct responses, ranging from 0 to 3 [86,96]. Secondly, descriptive statistics were conducted. Proportions and percentages were used to describe categorical data. Mean and standard deviation was reported to present normally distributed continuous data, while non-normally distributed data median and interquartile range (IQR) were presented. Respondents were segmented based on their MFRL applying k-means segmentation. Profiling of the clusters according to MFRL dimensions was assessed using logistic regression. Moreover, a comparison of sociodemographic and lifestyle characteristics between the segments was performed employing the ANOVA, Chi-Square test, and Kruskal-Wallis H test.

Thirdly, factor analysis, reliability, validity (both convergent and discriminant), and multicollinearity of the proposed TRA–extended model constructs were determined in conjunction with partial least squares structural equation modelling (PLS-SEM) in WarpPLS software version 7.0. A default outer model algorithm PLS regression routine with a default inner model analysis (Warp 3 algorithm) and the bootstrapping resampling method (number of data resamples = 999) was utilised to test the research hypotheses H1–H12. The underlying assumption of the model was based on the original TRA model (i.e., a direct path from attitudes and subjective norms to intention, and thereby from intention to behaviour). The model fit was reported by the eight goodness-of-fit measures: average path coefficient, average r-squared values, average variance inflation factors (AVIF), average full collinearity variance inflation factor (AFVIF), Tenenhaus goodness-of-fit, Sympson’s paradox ratio, statistical suppression ration, and nonlinear bivariate causality direction ratio.

## 4. Result

Table 2 shows the profiling of the segments. Four consumer segments were generated from MFRL instruments employing k-mean clustering analysis. To retain the four segments, different starting values and different starting numbers of segments were applied, including a distance measure between the data points for estimation. The clusters might not be directly comparable given the differences in the MFRL items used, the four segments corresponded to “Conservative–28.47% of the sample”, “Adventurous–14.78%”, “Uninvolved–11.73%”, and “Eco-moderate–45%”, according to [75]. Conservative consumers were characterised by their strong interest in food involvement value, while the Adventurous were characterised by their interest in food innovation. Further, consumers in the Uninvolved segment were characterised by their low scores on the MFRL dimensions, while the Eco-moderate by their strong interest in food responsibility and convenience with average scores on food involvement, food innovation, and price.

Table 3 shows the sociodemographic and lifestyle characteristics of the sample which shows 72.4% of the participants were females. The majority of the participants (42.6%) were students, living alone (26.1%) with a bachelor’s degree (30.7%) following an omnivore dietary pattern (61.1%) and purchasing surplus meals very often (27.6%). About three-fourths of the respondents had bought surplus meals (76.3%), and among them, only 7% buy surplus meals daily. Sociodemographic and lifestyle characteristics (except for age, living status, and employment) differ significantly between the four segments.

Table 4 shows the respondents’ stated buying preferences for surplus meal types and the reason. The majority of the sample stated that they have no meal preference (37%), followed by plant-based (30%) and meat-based surplus meals (28.3%). The value provided (median and interquartile range) shows that the main reason for buying preferences towards meat-based surplus meals includes liking to eat meat (4[1]) and it provides more energy (4[1]), while the main for buying preference towards plant-based surplus meal includes liking eating vegetables (4[1]), has lower carbon footprint (4[1]), and is healthier (4[1]). The respondent who generally liked surplus meals stated no preference towards either meat or plant-based meal (4[1]). The main reason for being uninterested in buying surplus meal includes preferring to cook (5[1]) and not trusting the sensory attributes of surplus meals (5[1.5]).

The results from Table 5 show that all items measuring the TRA constructs and additional constructs of environmental concern, and MFRL dimensions loaded highly on the pre-determined factors–normalised structure loadings of the items of each construct were above 0.65 and significantly associated with the loadings of items in their respective constructs (*p* < 0.05). Thus, indicating acceptable convergent validity. Both the value of Cronbach’s alpha and composite reliability was above 0.70, indicating an acceptable homogeneity among the items of a respective construct as well as an acceptable construct’s reliability. The value of average variance extracted (AVE) was above the minimum threshold of 0.50 and the value of variance inflation factor (VIF) was below the maximum threshold of 3.3, indicating convergent validity and no multicollinearity among the constructs exists, respectively.

Table 6 presents the correlation between the constructs, the square root of the AVE, and descriptive statistics of the constructs. The result indicated that the square root of the AVE of each construct was greater than the inter-construct correlation coefficient and the inter-construct correlation coefficient was less than 0.8, confirming the discriminant validity of the constructs. The result reveals a significant relationship between the behaviour to buy surplus meals and other variables including attitudes, subjective norms, environmental objective knowledge, environmental concerns, responsibility, convenience, and intention.

### 4.1. Goodness-of-Fit Statistics

Table 7 shows model goodness-of-fit statistics. The result indicated that including environmental knowledge, environmental concern, and background factors in the original TRA model has a better predictive power of behaviour (R^2^ = 0.15) than the original TRA model and extended TRA model with environmental objective knowledge and environmental concern (R^2^ = 0.063). Further, the proposed TRA-extended model represented a good model fit (AVIF = 1.247, AFVIF = 1.475, Tenenhaus goodness-of-fit = 0.440, Sympson’s paradox ratio = 0.889, statistical suppression ratio = 0.778, nonlinear bivariate causality direction ratio = 0.931). Thus, the TRA extended model with the inclusion of environmental objective knowledge, environmental concern, and background factors was retained for PLS-SEM analysis.

### 4.2. Path analysis through PLS-SEM

Table 8 shows the results from the PLS-SEM analysis. The result indicated that attitudes and subjective norms were significant predictors of the consumers’ buying intention towards surplus meals to predict buying behaviour, thus supporting hypotheses, H1, H2, and H3. Attitudes were the main predictor of behavioural intention (β = 0.618, s.e = 0.037, *p* < 0.001), followed by subjective norms (β = 0.171, s.e = 0.036, *p* < 0.001). Behavioural intention (β = 0.239, s.e = 0.049, *p* < 0.001) significantly influences surplus meal buying behaviour. Further, environmental objective knowledge (β = 0.277, s.e = 0.046, *p* < 0.001) significantly influences consumers’ environmental concern to further influence attitudes towards surplus meal (β = 0.124, s.e = 0.072, *p* = 0.042), supporting hypotheses H5 and H6. Environmental objective knowledge (β = 0.083, s.e = 0.109, *p* = 0.222) had no significant influence on attitudes, while environmental concern had a direct influence on behavioural intention (β = 0.085, s.e = 0.039, *p* < 0.001), thus rejecting hypothesis H4 while supporting hypothesis H7.

Table 9 shows the results of the background factors (both MFRL factors as well as sociodemographic and lifestyle characteristics) that influence the constructs (attitudes, subjective norms, and behaviour) of the proposed TRA-extended model. Attitudes, subjective norms, and behaviour were significantly influenced by multiple background factors. Specifically, attitudes were influenced by innovation (β = 0.254, *p* < 0.001), indicating that consumers with high scores in food innovation have a more positive attitude towards surplus meals, supporting hypothesis H8c. Further, attitudes were influenced by gender (β = −0.074, *p* = 0.046), living status (β = 0.076, *p* = 0.025), education (β = −0.106, *p* = 0.011) and dietary pattern (β = 0.093, *p* = 0.018), supporting hypotheses H10b, H10c, H10d and H10g, respectively. The result indicated that favourable attitudes were noted among female consumers those living alone with a higher education attainment and following an omnivore dietary pattern. Subjective norms were influenced by age (β = −0.092, *p* = 0.049), living status (β = −0.084, *p* = 0.035), education (β = −0.092, *p* = 0.033) and employment status (β = −0.102, *p* = 0.045), supporting hypotheses H11a, H11c, H11d, and H11e, respectively. This indicated that older adults those not living alone with a high education attainment and who have full-time employment were influenced by social norms to purchase surplus meals. Surplus meal buying behaviour was influenced by involvement (β = −0.143, *p* = 0.003), responsibility (β = 0.113, *p* = 0.018), convenience (β = −0.136, *p* = 0.002), gender (β = 0.097, *p* = 0.018) and education (β = −0.104, *p* = 0.013), supporting hypotheses H9a, H9b, H9d, H10b, and H10d, respectively. This indicated that male consumers with higher education attainment who focus more on food responsibility and has lower food involvement and convenience stores had a higher buying frequency of surplus meal.

## 5. Discussion and Implication

This study aimed to understand consumers´-buying behaviour towards surplus meals employing the TRA framework extended with environmental objective knowledge, environmental concerns, MFRL, and sociodemographic and lifestyle factors. The result from the study shows that attitudes, subjective norms, and environmental concerns significantly influence buying behaviour towards surplus meals, mediated by behavioural intention. Especially, attitudes towards surplus meals were a strong and significant predictor of behavioural intention to buy surplus meals which are in line with previous studies conducted in Belgium, Switzerland, and Malaysia where attitudes were a significant predictor for green and sustainable food consumption [97,98,99]. Persson [100] argues that a person’s attitudes influence their view and belief about the food they eat and play an inescapable part in their ability to consume certain foods. Thus, targeting each consumer segment by designing a specific strategy, for instance, communicating food waste or providing information on the sensory quality of surplus meals might enhance a positive attitude toward buying behaviour [99].

Subjective norm was also a significant predictor of the intention to buy surplus meals that are aligned with previous studies [55,97,101]. People will be more likely to do what a rising number of people appear to be doing, especially, in a canteen setting where colleagues can have a significant impact on a person’s ethical behaviour through social pressure and the development of intention [102]. This means when a colleague is outspoken about environmental issues they can help turn that concern into a social norm [71]. Therefore, implementing intervention following the strategies of social norms marketing approach, for instance, media, posters, and word-of-mouth could be used to publicise sustainable food consumption that might influence consumers to buy surplus meals in the canteen [103,104].

Moreover, environmental concern was a significant predictor of attitude towards surplus meals as well as intentions and is consistent with previous consumer studies that predicted intention to purchase green products [65,71]. The result of this study confirms that consumers’ environmental concerns indirectly influence their intention through their attitude toward surplus meals [105]. Thus, designing interventions to promote information on how buying surplus meals can benefit the environment by reducing food waste is recommended.

Environmental objective knowledge had no significant influence on attitude; however, it had a significant influence on environmental concern. The results are inconsistent with the finding from previous studies where environmental objective knowledge had a significant influence on attitudes toward green consumption [64,66]. According to the author of [106] consumers recognizes the importance of the environment; however, that does not necessarily translate into their level of environmental objective knowledge. In general, consumers are overconfident about themselves, resulting in higher subjective knowledge than objective knowledge [107]. This might explain Danish consumers´ positive attitude toward buying surplus meals due to environmental concerns without having enough objective knowledge about environmental issues. Moreover, growing environmental concern drives consumers to favour sustainable consumption. Therefore, practitioners should develop programs to enhance consumers’ environmental concerns and create a positive attitude toward the purchase of surplus meals and this could be achieved by providing the right environmental knowledge [108].

The intention is a significant but weak predictor of behaviour indicating discrepancies between behavioural intention and surplus meal buying behaviours. The results are consistent with previous findings on sustainable food consumption [109] and food waste behaviour [110]. Vermeir and Verbeke [109] highlight that in real-life situations, many other factors may have a role in actual purchase decisions. For instance, availability, situational and product-related factors may have a significant role in addition to other individual traits.

MFRL factor, food innovative was positively impacting Danish consumers’ attitude toward surplus meals; however, their behaviour was influenced by a strong sense of responsibility with the food. The results from this study also suggest that the food innovativeness score was high among the Adventurous consumer segment, while the food responsibility score was high among the Eco-moderate segment. However, this does not imply that the Eco-moderate consumer segment has higher surplus meals buying behaviour as the segment had also a high convenience score. The finding is in line with previous research among Danish consumers where innovativeness with the food was reported to be influencing attitudes [111]. Interestingly, the finding from this study shows that consumers who focus on convenience had a lower buying frequency of surplus meals. The finding does not align with the previous research on green food consumption where consumers with high convenience scores resulted in higher green food consumption behaviour [112]. Food convenience could mean less effort in preparing meals for some whereas others may associate it with the quality of the food, therefore it could be attractive for consumers depending on their situation [113]. Consumers could associate different quality dimensions such as sensory quality, nutritional value, food safety issues, and other risks when it comes to buying surplus meals at the canteen [114]. For instance, the unavailability of proper storage of surplus meals (fridging option) and other practicalities could hinder consumers to buy surplus meals at the canteen even though they are convenience-oriented.

Sociodemographic and lifestyle factors (age, gender, living status, education, employment, and dietary pattern) seem to indirectly influence intention by their effects on attitudes, and subjective norms, whereas gender and education seem to directly influence buying surplus meal behaviour. However, consumers with an education level of bachelor’s or above reported positive attitudes and social pressure and had a higher buying behaviour. Consumers with full-time employment, aged more than 25 years who were not living alone reported social pressure to purchase surplus meals. Female consumers reported positive attitudes whereas male consumers seemed influenced by social norms and had higher buying behaviour. A previous study on organic food buying behaviour reported similar findings on age and education [81]. Further, gender, age, and employment status were significant predictors of food waste behaviour in Denmark [115]. Further, consumers following omnivore dietary patterns reported favourable attitudes to buy a surplus meal. The finding is inconsistent with a previous study conducted in Denmark where the dietary pattern had no association with sustainable consumption behaviour [86].

The study has some limitations. The study applied a cross-sectional design, thus, limiting the ability to make causal inferences between the constructs of the proposed TRA-extended framework. The study employed a convenience sampling technique that could limit the generalization of the present findings. The sample was biased in terms of young female consumers but understanding them might be of importance for marketers as they represent a relevant target group for targeting change in buying behaviour. Moreover, the comparison and contrast of the findings are limited to research on sustainable food consumption, green consumption, and food waste behaviour due to limited previous studies on surplus meal buying behaviour. The study used environmental objective knowledge to predict surplus meal buying behaviour; however, including subjective knowledge could have more predictive power and provide a multi-dimensional understanding of environmental knowledge [116]. Moreover, the study employed a brief measure of MFRL to minimize the participant burden and thus may have limited more precise segmentation of consumers. Lastly, including other factors such as situational factors (availability), and product-related factors, could have strengthened the understanding of consumers’ decision-making process regarding surplus meal purchases [109]. Lastly, the surplus meal buying behaviour was measured with one item that may have resulted in methodological challenges [117,118].

## 6. Conclusions

In conclusion, this study identified Danish consumer segmentation based on their food-related lifestyle that comprised four segments: “Conservative”, “Adventurous”, “Uninvolved”, and “Eco-moderate”. Consumers´ buying behaviour towards surplus meal were analysed using the extended TRA framework. The results indicated that attitudes and subjective norms were significantly influencing behavioural intention to eventually influence buying behaviour. The environmental objective knowledge was significantly influencing environmental concerns, which further influenced attitudes and behavioural intention. However, environmental objective knowledge had no significant influence on attitudes. MFRL, sociodemographic, and lifestyle characteristics were found to be influencing behavioural intention indirectly by their effect on attitude and subjective norms. More favourable attitudes were noted among female consumers those living alone with a higher education attainment and following an omnivore dietary pattern. While older adults who were not living alone with a high education attainment and who had full-time employment perceived social pressure to purchase a surplus meal. Food involvement, food responsibility, convenience, gender, and education had a direct influence on surplus meal-buying behaviour, indicating that male consumers with higher education attainment who focus more on food responsibility and had lower food involvement and convenience stores had a higher buying frequency of surplus meals.

Future studies could apply other behavioural theories when surplus meals are more commonly available in Danish canteens, for instance, the theory of planned behaviour to understand consumers´ perceived behavioural control influence on behavioural intention. Future studies may focus on examining how situational factors such as the availability of surplus meals in canteens could explain the discrepancies between intention and actual buying behaviour. In future, intervention studies targeting food waste behaviour among young consumers should consider more context-specific strategies as young consumers are such a unique target group who are most likely to waste food [119,120,121] as well as engage in food waste reduction behaviour [122,123,124].

## Figures and Tables

**Table 1:** Items measuring the constructs of the proposed TRA-extended model and MFRL instrument.

Constructs	Items	Source of Adaption
Attitude (ATT)	ATT1: Buying surplus meals makes me feel good. ATT2: I think buying canteen surplus meals is environmentally friendly. ATT3: I think buying surplus meals in the canteen will save money for me compared to buying normal takeaway/ready-to-go meals.	[86]
Subjective norm (SBN)	SBN1: People who are important to me support that I buy surplus meals in the canteen. SBN2: People who are important to me think that I should buy surplus meals in the canteen. SBN3: I let the opinion of people who are important to me determine whether I will buy. surplus meals in the canteen or not.	[87,88]
Environmental concern (ENC)	ENC1: Climate change is happening. ENC2: The effort to reduce climate change is urgent.	[89,90]
Environmental Objective Knowledge (EOK)	EOK1: The contribution of food wastage emissions to global warming is almost equivalent to global road transport emissions. EOK2: Approximately one-third of edible food produced for human consumption is wasted or lost globally. EOK3: Animal-based products have higher carbon emissions than plant-based products.	[10,91]
Intention	INT1: I am willing to buy surplus meals in the canteen if they are available. INT2: I plan to buy food surplus meals in the canteen if they are available.	[88]
Behaviour	BEH: How often do you buy surplus meals?	[86]
Food Involvement (FIV)	FIV1: Eating and food are an important part of my social life. FIV2: Decisions on what to eat and drink are very important to me.	[75,92,93]
Food Responsibility (FRP)	FRP1: I try to choose food produced with minimal impact on the environment. FRP2: It is important to understand the environmental impact of our eating habits.	
Food Innovation (INN)	INN1: I like to try new food that I have never tasted before.	
Convenience (CON)	CON1: I use a lot of ready-to-eat foods in our household. CON2: To me, the microwave oven is essential for my cooking.	

**Table 2:** Profiling of clusters by MFRL instruments ^a^.

Total: n = 460	Segment 1 Conservative (n = 131)		Segment 2 Adventurous (n = 68)		Segment 3 Uninvolved (n = 54)		Segment 4 Eco-Moderate (n = 207)	
	OR	CI	OR	CI	OR	CI	OR	CI
Food Involvement	**11.292**	**6.151–20.728**	**0.022**	**0.008–0.059**	0.814	0.372–1.780	**1.873**	**1.210–2.8899**
Food Responsibility	**0.101**	**0.057–0.177**	**2.429**	**1.352–4.362**	1.785	0.644–4.950	**29.687**	**13.791–63.906**
Convenience	**0.067**	**0.037–0.123**	**2.184**	**1.322–3.611**	1.293	0.536–3.119	**23.211**	**11.745–45871**
Food Innovation	1.156	0.818–1.634	**6.774**	**3.379–13.579**	**0.002**	**0.000–0.014**	**4.191**	**2.617–6.712**
Price	**1.963**	**1.341–2.874**	**0.227**	**0.138–0.373**	**0.148**	**0.059–0.375**	**2.244**	**1.482–3.397**

^a^ Bold numbers indicate significant odd ratios, OR = odds ratio, CI = confidence interval.

**Table 3:** Sociodemographic and lifestyle factors of the four segments and their differences.

		**Conservative** **n = 131** **% (n)**	**Adventurous** **n = 68** **% (n)**	**Uninvolved** **n = 54** **% (n)**	**Eco-moderate** **n = 207** **% (n)**	**Total n = 460** **% (n)**	***p*-Value**
Age, y, median (IQR)		26 (13)	25.5 (17.75)	30 (21.25)	27 (15)	27 (15)	0.725 ^a^
Gender	Male	16 (21)	39.7 (27)	29.6 (16)	30.4 (63)	27.6 (127)	0.002 **^b^
	Female	84 (110)	60.3 (41)	70.4 (38)	69.6 (144)	72.4 (333)	
Living status	Alone	24.4 (32)	27.9 (19)	33.3 (18)	24.6 (51)	26.1 (120)	0.538 ^b^
	With partner	30.5 (40)	20.6 (14)	13 (7)	24.2 (50)	24.1 (111)	
	With a partner and children	14.5 (19)	20.6 (14)	25.9 (14)	23.7 (23)	20.9 (96)	
	Single parent	1.5 (2)	1.5 (1)	3.7 (2)	2.4 (5)	2.2 (10)	
	Living at home with my parents	10.7 (14)	14.7 (10)	13 (7)	8.7 (18)	10.7 (49)	
	Living with roommates	16.8 (22)	11.8 (8)	11.1 (6)	14.5 (30)	14.3 (66)	
	Other	1.5 (2)	2.9 (2)	-	1.9 (4)	1.7 (8)	
Employment	Student	45 (59)	38.2 (26)	38.9 (21)	43.5 (90)	42.6 (196)	0.156 ^c^
	Full-time job	42.7 (56)	30.9 (21)	35.2 (19)	39.1 (81)	38.5 (177)	
	Other	3.5 (16)	30.9 (21)	25.9 (14)	17.4 (36)	18.9 (87)	
Education	Primary school	2.3 (3)	2.9 (2)	7.4 (4)	1.4 (3)	2.6 (12)	0.016 *^c^
	High school	9.2 (12)	14.7 (10)	7.4 (4)	8.2 (17)	9.3 (43)	
	Vocational training	9.2 (12)	11.8 (8)	7.4 (4)	2.9 (6)	6.5 (30)	
	Professional bachelor	8.4 (11)	13.2 (9)	11.1 (6)	8.7 (18)	9.6 (44)	
	Bachelor	29 (38)	25 (17)	31.5 (17)	33.3 (69)	30.7 (141)	
	Master	35.1 (46)	19.1 (13)	18.5 (10)	26.1 (54)	26.7 (123)	
	PhD	0.8 (1)	4.4 (3)	1.9 (1)	4.3 (9)	3 (14)	
	Other	6.1 (8)	8.8 (6)	14.8 (8)	15 (31)	11.5 (53)	
Income	Less than 100,000	28.2 (37)	32.4 (22)	27.8 (15)	43 (89)	35.4 (163)	0.009 **^c^
	100,000–249,999	28.2 (37)	29.4 (20)	31.5 (17)	29.5 (61)	29.3 (135)	
	250,000–499,999	26.7 (35)	20.6 (14)	22.2 (12)	14.5 (30)	19.8 (91)	
	500,000–649,999	6.9 (9)	8.8 (6)	11.1 (6)	6.3 (13)	7.4 (34)	
	More than 650,000	9.9 (13)	8.8 (6)	7.4 (4)	6.8 (14)	8 (37)	
Dietary pattern	Omnivore	72.5 (95)	58.8 (40)	70.4 (38)	52.2 (108)	61.1 (281)	0.026 *^b^
	Flexitarian	12.2 (16)	25 (17)	18.5 (10)	26.6 (55)	21.3 (98)	
	Vegetarian	4.6 (6)	4.4 (3)	5.6 (3)	7.7 (16)	6.1 (28)	
	Vegan	10.7 (14)	11.8 (8)	5.6 (3)	13.5 (28)	11.5 (53)	
Surplus meal preference	Meat-based	33.8 (44)	17.7 (23)	13.8 (18)	34.6 (45)	28.3 (130)	0.013 *^b^
	Plant-based	22.5 (31)	13 (18)	10.1 (14)	54.3 (75)	30 (138)	
	No preference	30 (51)	14.1 (24)	8.8 (15)	47.1 (80)	37 (170)	
	I don’t want to buy a surplus meal	22.7 (5)	13.6 (3)	31.8 (7)	31.8 (7)	4.8 (22)	
Purchase frequency	Never	7.8 (36)	3.9 (18)	2.8 (13)	9.1 (42)	23.7 (109)	0.021 *^c^
	Rarely	4.8 (22)	2.6 (12)	2.2 (10)	5 (23)	14.6 (67)	
	Sometimes	8.9 (41)	3.5 (16)	2.6 (12)	12.2 (56)	27.2 (125)	
	Very often	6.1 (28)	3.5 (16)	2.8 (13)	15.2 (70)	27.6 (127)	
	Daily	0.9 (4)	1.3 (6)	1.3 (6)	3.5 (16)	7 (32)	

^a^ ANOVA, ^b^ Chi-square, ^c^ Kruskal Wallis H, * Significant effect at *p* < 0.05, ** significant effect at *p* < 0.01.

**Table 4:** The respondent’s buying preferences towards surplus meal types and the reasons, N = 460.

Surplus Meal Preference	% (n)	Reasons	Median	IQR
Meat-based	28.3 (130)	I like eating meat	4	1
		It can avoid producing more carbon emissions	3	2
		It is healthier than plant-based surplus meals	3	2
		It can provide more energy	4	1
Plant-based	30 (138)	I like eating vegetables	4	1
		It has a lower carbon footprint	4	1
		It is healthier than meat-based surplus meals	4	1
		I am a vegetarian/vegan	4	2
		It is cheap to buy a more plant-based surplus meal	4	2
No preference	37 (170)	…if I like it	4	1
		…if it is cheap	3	2
		…if I do not need to cook myself	3	2
		…if my actions can reduce my carbon footprint	3	1.25
I don’t want to buy a surplus meal	4.8 (22)	I do not want to eat the same meals in a row	4	2.25
		I prefer to cook	5	1
		I do not trust the sensory attributes of surplus meals	5	1.5
		I do not trust the food safety of surplus meals	4	2
		I feel ashamed buying surplus meals	2	2.5
		I do not want to pay for a surplus meal	4	2
		It is too complicated to buy and bring it home	4	3

IQR = Inter Quartile Range, reasons scored in 5–point Likert scale ranging from ‘strongly disagree’ to ‘strongly agree’.

**Table 5:** Confirmatory factor analysis, validity, reliability, and multicollinearity tests.

Constructs	Items	Normalised Structure Loadings	Cronbach’s Alpha	AVE	CRC	VIF
Attitude (ATT)	ATT1 ATT2 ATT3	0.656 0.722 0.725	0.847	0.766	0.908	2.239
Subjective norms (SBN)	SBN1 SBN2 SBN3	0.736 0.762 0.744	0.748	0.677	0.859	1.432
Environmental concern (ENC)	ENC1 ENC2	0.765 0.711	0.836	0.859	0.924	1.857
Food Involvement (FIV)	FIV 1 FIV2	0.807 0.802	0.733	0.790	0.882	1.254
Food Responsibility (FRP)	FRP1 FRP2	0.725 0.715	0.789	0.826	0.904	1.850
Convenience (CON)	CON1 CON2	0.924 0.916	0.722	0.782	0.878	1.248
Intention (INT)	INT1 INT2	0.712 0.702	0.913	0.920	0.958	2.234

AVE = average variance extracted, CRC = composite reliability, VIF = variance inflation factor.

**Table 6:** Descriptive statistics and correlation among the constructs with square roots of average variance extracted.

Constructs	ATT	SBN	EOK	ENC	FIV	FRP	CON	INN	INT	BEH
Attitude (ATT)	**0.875**									
Subjective norms (SBN)	0.368 ***	**0.823**								
Environmental objective knowledge (EOK)	0.094 *	0.157 *	**1**							
Environmental concern (ENC)	0.337 ***	0.064	0.217 ***	**0.927**						
Involvement (FIV)	0.208 ***	0.148 **	0.112 *	0.360 ***	**0.889**					
Responsibility (FRP)	0.250 ***	0.205 ***	0.344 ***	0.524 ***	0.233 ***	**0.909**				
Convenience (CON)	0.054	−0.175 ***	−0.084	0.121 *	−0.137 **	−0.121 **	**0.885**			
Innovation (INN)	0.322 ***	0.137 **	0.299 ***	0.100 *	0.271 ***	0.233 ***	−0.091	**1**		
Intention (INT)	0.709***	0.406 ***	0.074	0.309 ***	0.170 ***	0.246 ***	0.076	0.237 ***	**0.959**	
Behaviour (BEH)	0.172 ***	0.246 ***	0.128 **	−0.099 *	−0.070	0.122 **	−0.125 **	−0.017	0.209 ***	**1**
Mean	3.847	3.061	0.573	4.323	3.918	3.695	3.306	3.82	3.767	2.80
Standard deviation	0.872	0.868	0.356	0.747	0.799	0.863	1.085	0.965	1.00	1.269

* Significant effect at *p* < 0.05, ** significant effect at *p* < 0.01, *** significant effect at *p* < 0.001, the bold value represents the square root of average variance extracted (AVE).

**Table 7:** Goodness-of-Fit Statistics.

Model Goodness-of-Fit Statistics	Original TRA Model ^a^	TRA-Extended Model with EOK and ENC	TRA-Extended Model with EOK, ENC, and Background Factors	Standard Norms ^b^
Average path coefficient	0.356 ***	0.258 ***	0.107 ***	
Average R-squared	0.296 ***	0.199 ***	0.211 ***	
AVIF	1.158	1.148	1.247	≤3.3
AFVIF	1.627	1.511	1.475	≤3.3
Tenenhaus goodness-of-fit	0.499	0.417	0.440	large ≥ 0.36
Sympson’s paradox ratio	1.000	1.000	0.889	≥0.7
Statistical suppression ratio	1.000	1.000	0.778	≥0.7
Nonlinear bivariate causality direction ratio	0.833	0.929	0.931	≥0.7
R^2^ (Intention)	0.529	0.536	0.536	
R^2^ (Behaviour)	0.063	0.063	0.150	
Stone-Geisser Q-squared coefficient (Intention)	0.529	0.536	0.536	
Stone-Geisser Q-squared coefficient (Behaviour)	0.064	0.064	0.155	

^a^ direct path from attitudes and subjective norms to intention, and thereby from intention to behaviour [52,53], ^b^ TRA = theory of reasoned action, EOK = environmental objective knowledge, ENC = Environmental concern, AVIF = average variance inflation factors, AFVIF = average full collinearity variance inflation factor, *** significant effect at *p* < 0.001.

**Table 8:** Path analysis of the proposed TRA-extended model and its status.

Paths	Standardised (Beta) Coefficient	Standard Error	*p*-Value	Hypothesis Status
ATT to INT	0.618	0.037	***	H1: Supported
SBN to INT	0.171	0.036	***	H2: Supported
INT to BEH	0.239	0.049	***	H3: Supported
EOK to ATT	0.083	0.109	0.222	H4: Rejected
EOK to ENC	0.277	0.046	***	H5: Supported
ENC to ATT	0.124	0.072	0.042 *	H6: Supported
ENC to INT	0.085	0.039	0.014 *	H7: Supported

ATT = attitudes, SBN = subjective norms, EOK = environmental objective knowledge, ENC = environmental concern, INT = intention, BEH = behaviour. Significant codes: * = *p* < 0.05, *** = *p* < 0.001.

**Table 9:** Path analysis between background factors and constructs of the proposed TRA extended model.

	Endogenous Variables					
	H8 & H10: ATT		H11: SBN		H9 & H12: BEH	
R^2^	0.235		0.058		0.150	
MFRL factors	Coeff	*p*	Coeff	*p*	Coeff	*p*
(a) Involvement	0.122	0.087			**−0.143**	**0.003**
**(b)** Responsibility	0.076	0.081			**0.113**	**0.018**
**(c)** Innovation	**0.254**	***			−0.052	0.180
(d) Convenience	0.098	0.125			**−0.136**	**0.002**
Sociodemographic and lifestyle factors						
(a) Age (≤25 =1)	−0.055	0.150	**−0.092**	**0.049**	−0.004	0.471
(b) Gender (male = 1)	**−0.074**	**0.046**	0.021	0.338	**0.097**	**0.018**
**(c)** Living status (alone = 1)	**0.076**	**0.025**	**−0.084**	**0.035**	−0.037	0.192
(d) Education (≤bachelor = 1)	**−0.106**	**0.011**	**−0.092**	**0.033**	**−0.104**	**0.013**
**(e)** Employment (student = 1)	0.023	0.338	**−0.102**	**0.045**	0.027	0.318
(f) Income (≤100,000 = 1)	−0.043	0.171	0.023	0.326	0.070	0.071
(g) Dietary pattern (omnivore = 1)	**0.093**	**0.018**	0.004	0.462	−0.025	0.280

ATT = attitudes, SBN = subjective norms, BEH = behaviour. Significant codes: *** = *p* < 0.001, the bold value represents significant effect.

## Data Availability

The data presented in this study are available on request from the corresponding author.
